# Onco-Esthetics Dilemma: Is There a Role for Electrocosmetic-Medical Devices?

**DOI:** 10.3389/fonc.2020.528624

**Published:** 2021-01-29

**Authors:** Beniamino Palmieri, Lucia Palmieri, Andrea Mambrini, Valentina Pepe, Maria Vadalà

**Affiliations:** ^1^Department of General Surgery and Surgical Specialties, University of Modena and Reggio Emilia Medical School, Surgical Clinic, Modena, Italy; ^2^Second Opinion Medical Network, Modena, Italy; ^3^Nephrology, Dialysis and Transplantation Complex Structure, Hospital of Modena, Modena, Italy; ^4^Medical Oncology Department, ASL Toscana Nord Ovest, Carrara, Italy

**Keywords:** cancer, esthetics, oncology esthetics, medical devices, cosmetic, electro-cosmetic device, onco-esthetic, esthetician

## Abstract

**Objective:**

The primary aim of this review is to verify whether the warning against the use of electromedical instruments in the cosmetic professional or medical cancer patient settings is consistent with evident oncological risks supported by experimental *in vitro*/*in vivo* studies or anecdotal clinical reports, or any other reasonable statement.

**Methods:**

MEDLINE, PubMed, Embase, AMED, Ovid, Cochrane Controlled Trials Register, and Google Scholar databases were electronically searched. Data relating to research design, sample population, type of electro-cosmetic devices used, were extracted.

**Results:**

The search strategy identified 50 studies, 30 of which were potentially relevant.

**Conclusions:**

Our research is in favor of moderate periodical use of cosmetic medical devices in patients bearing tumors, in any stage, like in healthy people. Special consideration is dedicated to massage, manipulation, and pressure delivery upon the cytoskeleton of cancer cells that has proven to be sensitive to mechanical stress at least in some specific locally relapsing cancers such as osteosarcoma.

## Introduction

Genetic and epigenetic events define the evolution from normal cells to the malignant state, up to death. Tumor progression is determined by molecular mechanisms, almost completely identified, acting progressively step by step until the end of the life. In 2000, Hanahan and Weinberg developed the first 6 hallmarks of cancer that became 10 in 2011 (1).

The 10 cancer characteristics acquired by the cells during cancer development are:

(1) **Uncontrolled proliferation:** healthy cells actively control the growth cycle and cell division ensuring homeostasis and maintenance of tissue architecture and function. Cancer cells counteract these signals resulting in uncontrolled cell growth (1).
(2) **Altered metabolism:** the cancer cell metabolism is mainly based on aerobic glycolysis that breaks down glucose in aerobic conditions (1).
(3) **Apoptosis:** the healthy cells avoid aging and DNA/RNA mutations/deletions, with physiological death and regeneration; the cancer cells ignore this pathway.

(4) **Replicative immortality:** normal cellular aging is consistent with shortening of telomeres; cancer cells do not reduce their length.
(5) **Block of the onco-suppressor genes:** onco-suppressors have been discovered by experimental activation–inactivation in mice or human cancer tissues. They code for proteins that can inhibit and/or downstage the proliferation (1).
(6) **Genomic instability:** gene mutations and chromosome fragmentation, cross over, and partial loss, increase into cancerized nuclei. Normal cells counteract partially these damages activating their endogenous DNA repair system.
(7) **Inflammation:** cancer cells express very often inflammatory cytokines that help cancer growth.
(8) **Immune defenses:** the check point of immune control of normal cell reproduction is lost in the majority of malignant tumors.
(9) **Metastasis:** cancer cells can colonize far from their primary site through active cytoskeleton motility via adhesion receptors and coagulation–fibrinolytic interactions.
(10) **Angiogenesis:** newly formed blood vessels arise into and around the cancer masses to supply oxygen and nutrients and output carbon dioxide and waste catabolites (1).

**FIGURE 1:**
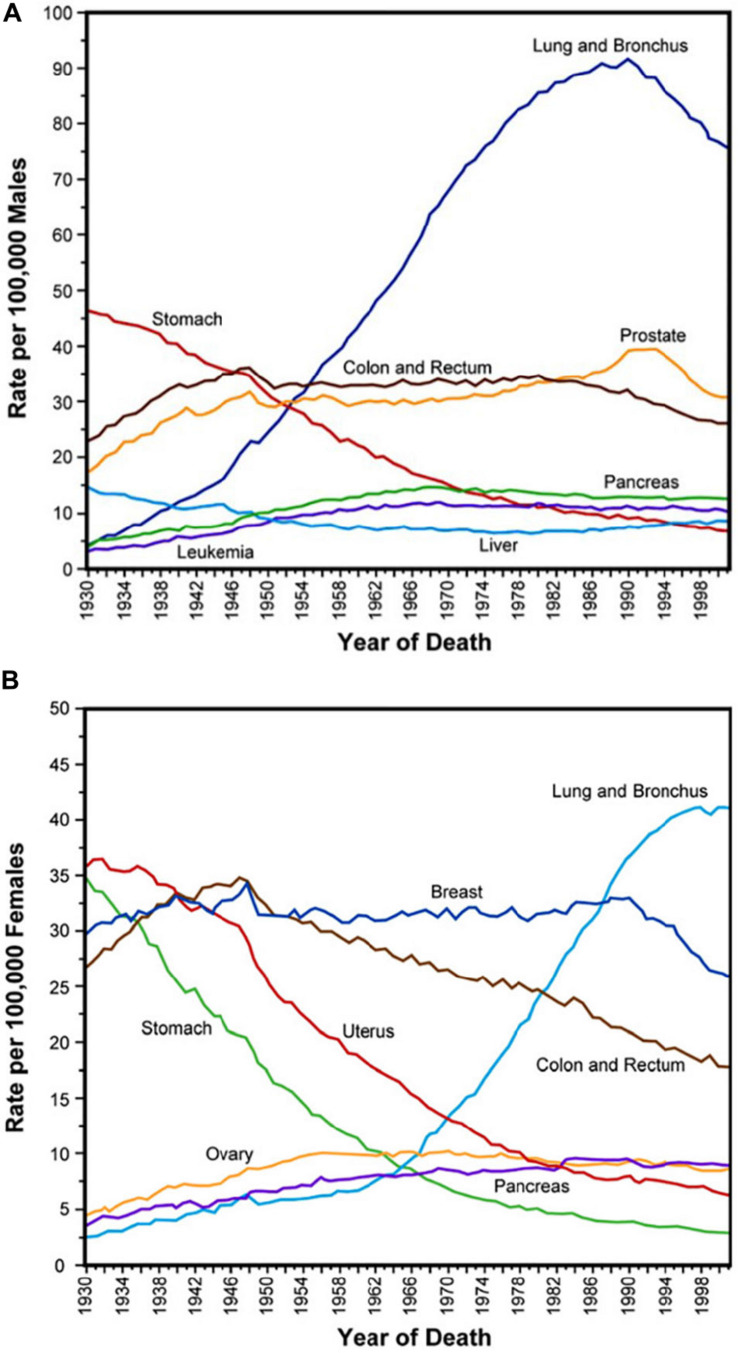
**(A,B)** Gender age-adjusted cancer mortality rates over seven decades since 1930. Adapted from Subra Suresh (Department of Materials Science and Engineering, Division of Biological Engineering, and Harvard-MIT Division of Health Sciences and Technology, Massachusetts Institute of Technology, Cambridge, MA). Copyright 2007 Acta Materialia Inc., Published by Elsevier Ltd., All rights reserved.

The study of cancer cell biology gave the chance of excellent therapeutic results in many tumor histotypes, increasing the patient’s life expectancy (Figures 1A,B); by prolonging both disease free or metastatic survival rate, these very effective successes introduced the urgent need of onco aesthetic performances to rescue self-image, relieve stress, depression, and anxiety, and to achieve a better personal and social life quality during and after the oncological tunnel.

### Cancer Cell Biology

The peculiar trait of cancer cells is pooling of DNA mutations, altering regulation, differentiation, maturation, and apoptosis. This imbalance is multifactorial due to:

• Geographical and environmental factors,
• Advanced age,
• Chronic inflammation;
• Genetic predisposition with hereditary mutations (less than 10% of patients) with dominant autosomal forms,
• Mismatch repair syndrome,
• Family shapes.

**FIGURE 2:**
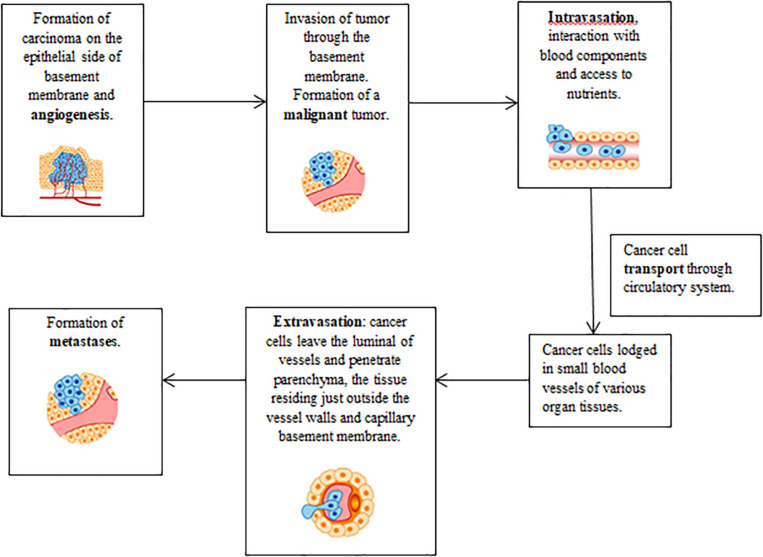
Steps involved in the cancer cell invasion–metastasis cascade. Adapted with modifications from Subra Suresh (Department of Materials Science and Engineering, Division of Biological Engineering, and Harvard-MIT Division of Health Sciences and Technology, Massachusetts Institute of Technology, Cambridge, MA). Copyright 2007 Acta Materialia Inc., Published by Elsevier Ltd., All rights reserved.

**FIGURE 3:**
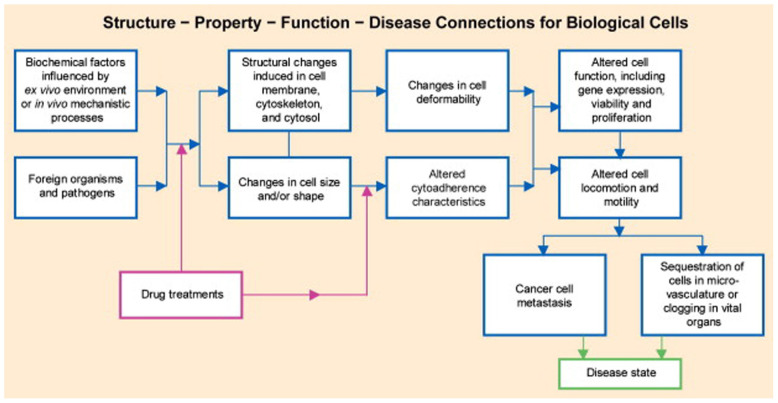
Schematic of chemo biomechanical pathways influencing connections among subcellular structure, cell biomechanics, motility, and disease state. This structure–function–disease paradigm in cancer is a new disease perspective. Copyright 2007 Acta Materialia Inc., Published by Elsevier Ltd., All rights reserved.

In natural conditions, the cells, after 50–60 reproductive cycles, undergo apoptosis, but in cancer cells, telomerase persists actively rendering them young and immortal. Exponential growth, accordingly, with the Gompertz’s law, is the result of continuous multiplication: later on, the cell turnover reaches a plateau, stabilizes, then slows down to balanced reproduction/versus death cycles. Viable cancer cells can rest quiescent for years either stopping the mitotic cycle or equally balancing proliferation and necrosis (Figures 2, 3).

### The Vascular System of Cancer

The growth, invasion, and formation of tumor metastases are induced by new vascularization, triggered by cancer cells through neo angiogenic cytokines (2). The newly formed microcirculation, whose inner lumen is partially outlined by endothelial healthy or cancer cells, connects and is intermingled with the preexisting normal vascular network, and through this escape way, they are allowed to migrate in the systemic blood stream.

Tumor angiogenesis is mediated by molecules or factors such as:

• Fibroblastic growth factor (FGF),
• Vascular endothelial growth factor (VEGF),
• Vascular permeability factor (VPF),
• Angiogenin,
• Epidermal growth factor (EGF).

### Inflammation and Cancer

**FIGURE 4:**
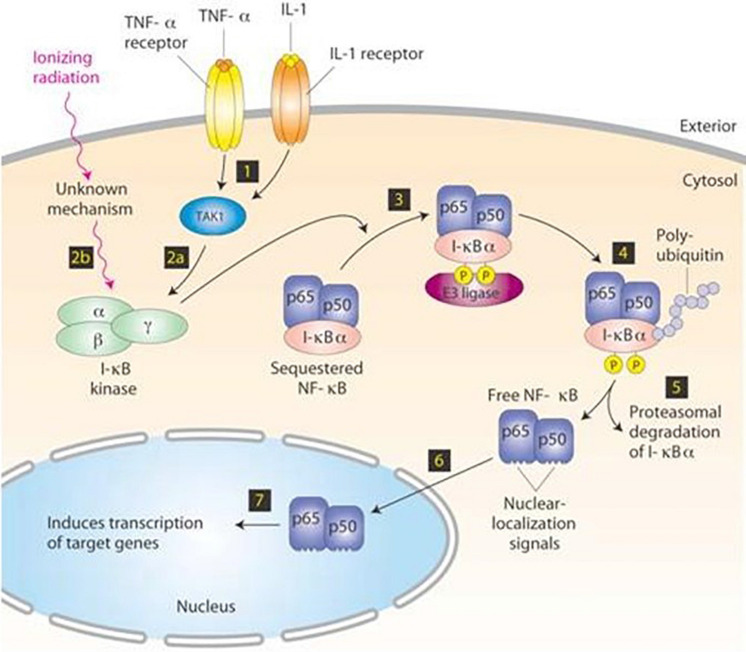
NF-kB signaling pathway. In resting cells, the dimeric transcription factor NF-kB, composed of p50 and p65, is sequestered in the cytosol, bound to the inhibitor I-kB. Stimulation by TNF-α or IL-1 induces activation of TAK1 kinase (step 1), leading to activation of the trimeric I-kB kinase (step 2a). Ionizing radiation and other stresses can directly activate I-kB kinase by an unknown mechanism (step 2b). Following phosphorylation of I-kB by I-kB kinase and binding of E3 ubiquitin ligase (step 3), polyubiquitination of I-kB (step 4) targets is for degradation by proteasomes (step 5). The removal of I-kB unmasks the nuclear-localization signals (NLS) in both subunits of NF-kB, allowing their translocation to the nucleus. Copyright 2011 Receptors and signal transduction Published by Technology. All rights reserved.

The tumor necrosis factor alpha (TNFα) is primarily engaged in host’s defense against bacterial and viral infection. Involved both in physiological and pathological processes, it causes cancer cell necrosis and apoptosis. Finally, TNF has emerged as an important risk factor for cancer progression, invasion, and metastases and is a key intermediary of chronic inflammation associated with cancer (3). It binds with two membrane receptors: TNF receptor 1 (TNFR1) and TNF receptor 2 (TNFR2) (3). The first receptor triggers the regulation system cascade such as NF-kB and some MAPKs (JNK, p38 and ERK), regulating immune and inflammatory responses (proinflammatory cytokines, chemokines, and adhesion molecules). NF-kB (Figure 4) is involved in the expression of apoptotic, molecules including c-FLIP and bcl2 family molecules (4).

### Complementary Therapies

Tumor pathology is obviously multifactorial, with variable role of genes, environment, nutrition, lifestyle (5, 6). Several investigations focused on the relationship between chronic stress and cancer development. Psychic factor plays a key role in tumor pathogenesis, treatment response, and prognosis. Recent studies evaluate the relationship between stress management in cancer patients and life quality/survival span rate (7–9). More recently, onco-aesthetics has been enclosed among the complementary therapies, added to manual and manipulative techniques, acupuncture, and mind–body therapies (yoga, meditation, oriental disciplines) (10). The beautician-patient or aesthetic-patient doctor relationships improve the self-image and patient satisfaction promoting an appealing social role previously deteriorated by the cancer history and treatments (11). The manual treatment is often integrated and empowered by the use of cosmetic electronic and electromedical devices, but there is a reluctant opinion about their utilization in cancer. In the present study, we have explored the state of the art in the medical literature about oncological safety and effectiveness of electromedical and cosmetic devices in the oncological area.

#### Manual Techniques: Massages

In the recent years, the use of complementary alternative medicine (CAM) has increased sharply, used as an adjuvant tool in oncological diseases and palliative care (12, 13). A very interesting Norwegian (14) study describes the prevalence of CAM in individuals with a previous cancer diagnosis. Men and women (12,982) filled a questionnaire about general health state, previous family and personal illnesses, pain and physical discomfort, food habits, alcohol consumption, smoking habits, physical activity in leisure time, level of education, and health services consultations including CAM. Younger, highly educated women (dominant young age) prevailed in the study. Globally 33.8% of all cancer survivors reported CAM use (39.4% of the women and 27.9% of the men).

The most common CAM massage techniques are:

• **Ayurvedic massage** restores the balance between mind and body in such a way that it can give the individual a psychophysical well-being.
• **Chinese Tui Na massage** relaxes muscles, improves bone system performance, and eliminates stress.
• **Do-in massage** is one of the best methods to combat stress.
• **Massage with acupressure** is effective especially in sleep disturbances.
• **Thai massage** releases energy and is supposed to promote detoxification.
• **Shiatsu massage** reduces stress and anxiety, promotes psychophysical relaxation, counteracts insomnia, and relieves ailments and joint pain.
• **Zonal massage** aids detoxification and reduces joint pain.

Aromatherapy (ATM), integrated with manual massage, is a very appealing approach in neuroaesthetics as assessed by Boehm et al. (15). His systematic review of preclinical and clinical studies shows evidence of anxiety and depression relief, controlling pain, improving sleep, muscle relaxation, and increased mood. The use of diluted essential oils has minimal risks in cancer patients. Prolonged topical administration might trigger some allergic or contact dermatitis. For this reason, the aromatherapy oncological patients should be actively followed by an expert dermatologist.

The mechanisms leading to benefits and recovery in the daily life are summarized as follows:

• Psychophysical relaxation; induced by the olfactory characteristics of aromatherapy.
• Childhood regression; determined by manual contact of the massage.
• Better perception of one’s own image.
• Restoration of the mind-to-body ratio.

**FIGURE 5:**
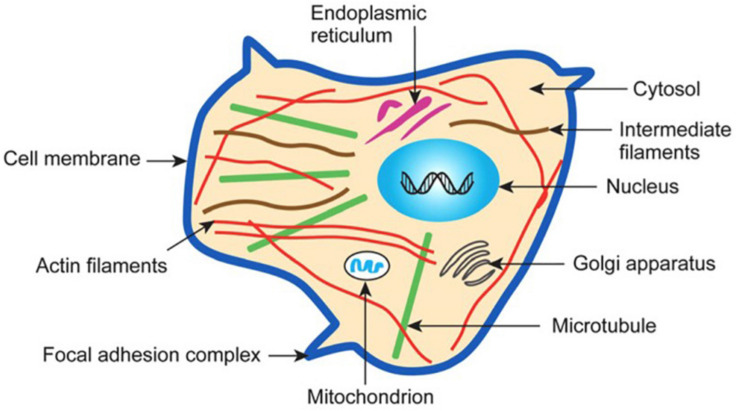
Schematic illustration of the subcellular structure of a typical eukaryotic cell. Adapted from Subra Suresh (Department of Materials Science and Engineering, Division of Biological Engineering, and Harvard-MIT Division of Health Sciences and Technology, Massachusetts Institute of Technology, Cambridge, MA). Copyright 2007 Acta Materialia Inc., Published by Elsevier Ltd., All rights reserved.

**FIGURE 6:**
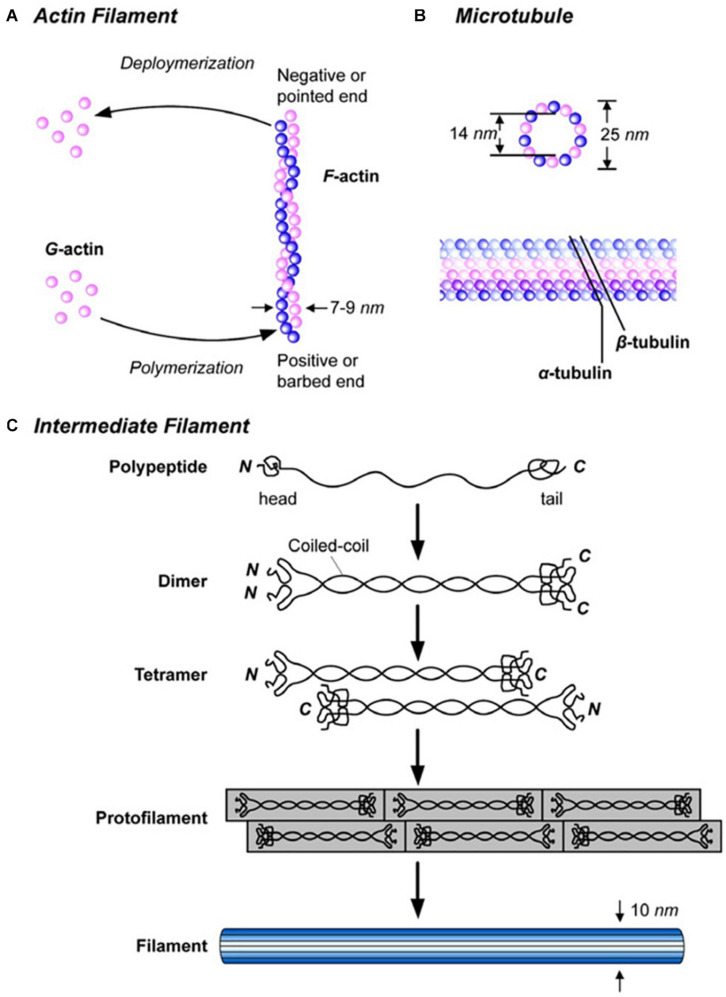
Description of the components of the cytoskeleton: structure of **(A)** actin microfilament (polymer that plays a key role in muscle contraction, cell movement and shape), **(B)** microtubule (long tubulin filament that plays a role in cell structure, organization, mitosis, and movement) and **(C)** intermediate filament (ropelike cytoskeletal filament that plays several different structural roles but is not involved in cell movement). Reproduced with permission from Subra Suresh (Department of Materials Science and Engineering, Division of Biological Engineering, and Harvard-MIT Division of Health Sciences and Technology, Massachusetts Institute of Technology, Cambridge, MA). Copyright 2007 Acta Materialia Inc., Published by Elsevier Ltd., All rights reserved.

**FIGURE 7:**
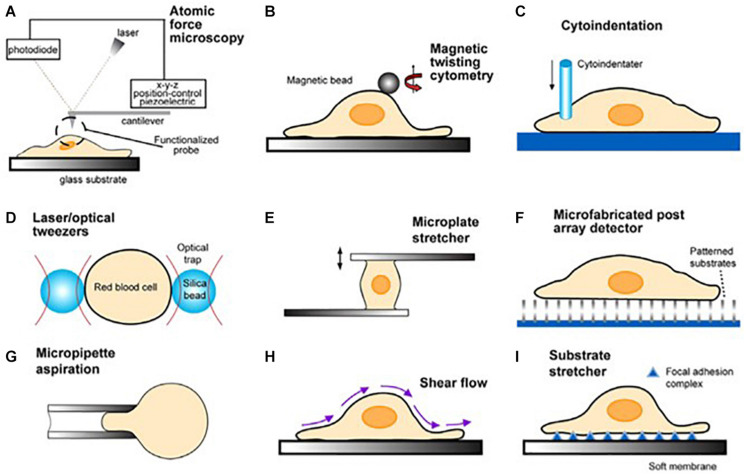
Schematic illustrations of the biomechanical assays on tumor cells **(A–C)**. Biophysical assays commonly used to probe the deformation of single cells are illustrated in **(D–G)**. Techniques used to infer cytoadherence, deformation, and mobility characteristics of populations of cells are schematically sketched in **(H**,**I)**. Image courtesy of, and with permission from, Subra Suresh (Department of Materials Science and Engineering, Division of Biological Engineering, and Harvard-MIT Division of Health Sciences and Technology, Massachusetts Institute of Technology, Cambridge, MA). Copyright 2007 Acta Materialia Inc., Published by Elsevier Ltd., All rights reserved.

**FIGURE 8:**
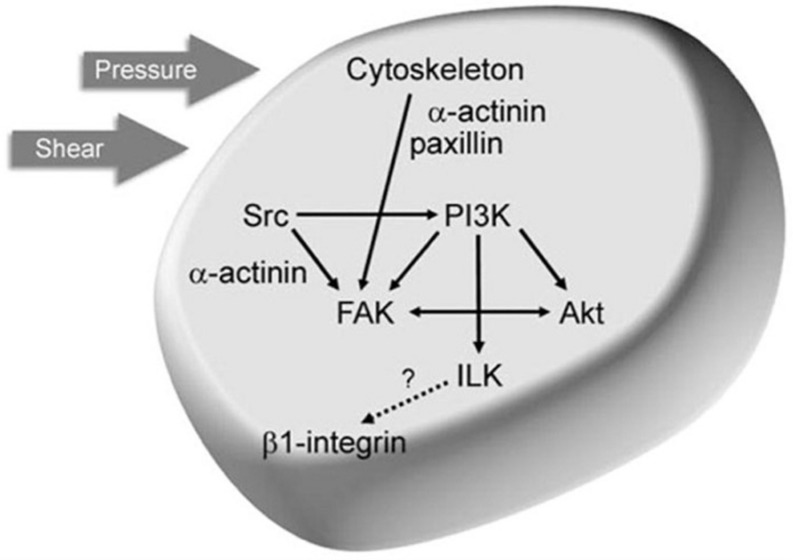
Working model of the mechanical response pathway by which pressure and shear stimulate tumor cell adhesion. FAK is activated in response to mechanical stimuli through a dual signaling pathway requiring an intact cytoskeleton, α-actinin, and paxillin as well as cytoskeleton-independent activation of Src. FAK and Src colocalize to β1-integrin heterodimers in an Akt- and α-actinin-dependent manner, respectively. FAK–Src complex formation and signaling stimulates PI3K-dependent activation of ILK, resulting in β1-integrin phosphorylation and conformational activation, increasing substrate binding affinity. Image courtesy of, and with permission from, Ref. David H. Craig (Department of Surgery; Michigan State University; Lansing, Michigan United States). Copyright 2009 Cell Cycle Published by HHS Public Access. All rights reserved.

Generally speaking, any kind of massage and manipulative technique must be performed by an experienced staff in order to avoid traumatic injuries. Adequate psychological approach to the subject is required as well to reach an excellent performance level. During the manual treatment, special care must be paid on the areas to be treated if the primary or secondary cancers are directly impending or borderline. Strong pressure in these areas might squeeze the blood vessels with reduced tumor perfusion. Compression of lymphatic vessels, on the other hand, hinders the tumor’s ability to drain excess fluid, increasing the interstitial fluid pressure. Both these hemodynamic consequences, hypoperfusion and interstitial hypertension, counteract the intratumor spreading of chemotherapeutic drugs, potentially reducing the effectiveness of the treatment. Prolonged hypoperfusion would induce a hypoxic and acid microenvironment indirectly, promoting tumor progression and metastasis (16). In a study by Wang et al. (17) on 235 patients with osteosarcoma (OS), the treatment with manipulative techniques (MT) leads to an unfavorable prognosis of primary osteosarcoma. In fact, many patients seek for manipulative therapy (MT), such as massage, Tuina, or other kinds of complementary treatments prior to diagnosis to relieve pain symptoms. This procedure is thus contraindicated. Previously, the same author had described an experimental *in vivo* model with MG-63 (human OS cells), turned to osteosarcomatous after GFP-lentivirus transfection, and then implanted, into the right and left joint (being the left used as control, without massage). The massage was repeated on the tumor site twice a week, not in the control group (MT-). The mice were sacrificed when the tumor size reached roughly the 2% of the body weight, and at the autopsy, bilateral legs, liver, lungs, lymph nodes, and serum were kept for immunohistochemistry. The rabbit anti-GFP antibody (1:1,000, Genetex, United States) marked MG-63 cells in the organs of tumor-transferred mice. The mice treated with MT had reduced survival with the highest recurrence rate or metastasis, accordingly with the human osteosarcoma study. Babahosseini et al. (18) showed in an ovarian cancer model that metabolites of bioactive sphingolipids are important lipid messengers, involved in generation and potentially modulation and control of some cancer. He demonstrated also that the elasticity of aggressive ovarian cancer cells decreased ∼15% after treatment with ceramide and sphingosine-1-phosphate. On the contrary, sphingosine treatment caused a ∼30% increase in the average elasticity, which was associated with a more defined actin cytoskeleton organization (Figures 5, 6). Recently, Brossel et al. (19) investigated the effects of biomechanical stress delivered across a tumor mass. The aim was to provide *in vivo* experimental evidence of influencing tumor growth by biomechanical signals. They inoculated in mice a human breast cancer cell line, MDA MB 231, mixed with subcutaneous ferric nanoparticles. These particles surrounded the tumor inducing a stiff constriction field (Figure 7). The administration of external mechanical loads upon the constricted tumor masses allowed tumor growth in the areas where the stress levels were lower. Cancer cell apoptosis and reduced proliferation rates were detected in the tumor surfaces surrounded by a strong belt of iron particles. Stylianopoulos (16) explains in his study how drugs that relieve solid and fluid stress could be used in combination with chemotherapy to improve the effectiveness of treatments. In his study, he considered separately the three main types of solid stress at the tissue level involved in tumor growth, namely, the externally exerted stress on the tumor from the host tissue, the stress swelling, and the residual stress. Hypoperfusion and interstitial hypertension counteract the uptake of anticancer drugs (even nanomedical agents) by the cancer tissues: in this way, high levels of mechanical stress can support the tumor progression. Further investigations about cancer cell cytoskeleton and their response to mechanical stimuli namely – pressure and shear forces (as it clinically happens during manipulations, manual or instrumental massage) showed that their integrin-binding affinity and intrinsic adhesion signaling system can be modified, the biological impact of the physical energies upon the natural history of cancer is currently deeply investigated, high pressure long time exposure promotes cancer cell multiplication through a cytoskeleton-dependent signaling mechanism requiring FAK, Src, Akt, and paxillin (Figure 8).

## Acupuncture

A 2010 Hui (20) study showed that stimulation of certain points through acupuncture determines the ability to activate some brain areas able to reduce sensitivity to pain and stress. It also promotes relaxation by disabling the “analytical brain” responsible for anxiety and worry. The mechanism of action of acupuncture has been studied with functional magnetic resonance imaging (fMRI). These studies defined that acupuncture stimulation, evaluated in the setting of *deqi* (scale of 10 sensations perceived by the patient during acupuncture: aching, soreness, pressure, fullness, heaviness, numbness, tingling, warmth, coolness, and dull pain, on a scale from 0 to 10), evokes deactivation of a limbic–paralimbic–neocortical network, up to the limbic system, as well as activation of somatosensory brain regions. The evidence-based studies show that the application of acupuncture reduces chemotherapy-induced nausea, leading to greater control of pain and anxiety. Last, but not least, the acupuncture is a highly safe and reliable integrative cancer therapy.

## Mind–Body Therapies

Mind–body therapies encompass meditation, hypnotherapy, yoga, tai chi, and music therapy, each one based on the synergy between brain, mind, body, and behavior. Tsai et al. (21) demonstrated that these techniques can reduce anxiety and stress levels improving life quality especially when used in combination with cancer drugs, meditation, and prayer, and reduce the fear of death by offering contemplation and faith support and helping to positively cope with the stress-related disease progression.

## Cosmetic–Medical Instruments

Each type of electromedical instrument is associated with a number of warnings and caution in the cosmetic oncological use, without any clear-cut evidence-based rationale. Intuitively, however, local or regional delivery of these energies, far from the tumor area, cannot be responsible of any interference with cancer growth or spread. Currently, the instruments that use low-scale, energy for onco aesthetic purposes are:

• Ultrasound (HIFU),
• Electroporation,
• Electromagnetic inductors,
• High- and low-frequency magnetotherapy,
• Heat-generating instruments such as radio frequency,
• Tecar,
• Electrotherapy,
• Cryotherapy.

These active principles, in our opinion, have no absolute contraindications in cancer because they release low energy across a very limited depth across (skin–dermis–subcutis) for the cosmetic restoration of epidermis collagen reticular and elastic fibers. Electromedical instruments that exploit vacuum therapy or activate venous, arterial, and lymphatic circulation (pressotherapy) are applied in places of the body where congestion and stasis are subsequently caused by wide lymph node excisions or dense collagen scars (like in the forearm post mastectomy lymphedema or “brass gross” or in heavily irradiated areas). Some experimental and clinical investigations focused on the cancer cells spreading risk through the endothelial gaps of the capillaries, resulting in metastatic progression. Although this hypothesis may be supposedly theoretically reasonable, the huge, complex network of receptors, adhesive systems, and coagulation fibrinolysis, and permeability factors exclude that low power energies have any direct independent chance to promote instant growth and dissemination of cancer. In the Craig’s (22) study, exposure of cancer cells to increased extracellular pressure has been shown to cause:

• adhesion of cancer cells to matrix proteins;
• stimulates the proliferation of cancer cells.

In a murine model of Co26 and Co51 cancer cells implanted into a surgical wound, the burden of 15 mmHg pressure on tumor cells reduced the survival rate of the animals, in comparison with cells under normal pressure or pretreated with colchicine. Cancer lines from the colon and breast express the same behavior through adhesion to matrix proteins, endothelial cell monolayers, and experimental surgical wound margins with intrinsic signal variations in integrin-binding affinity.

**FIGURE 9:**
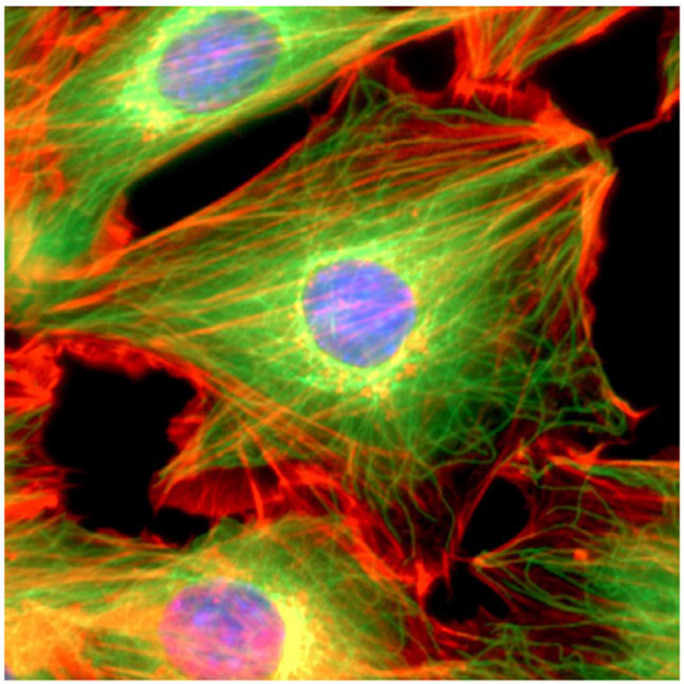
A mouse NIH3T3 fibroblast cell was fixed and stained for DNA (blue) and the major cytoskeletal filaments actin (red) and alpha-tubulin (green). The cell was imaged by fluorescence microscopy on an optical IX70 microscope with a deep-cooled CCD camera. Image courtesy of, and with permission from, Andrew E. Pelling (London Centre for Nanotechnology and Department of Medicine, University College London). Copyright 2007 Acta Materialia Inc., Published by Elsevier Ltd., All rights reserved.

**FIGURE 10:**
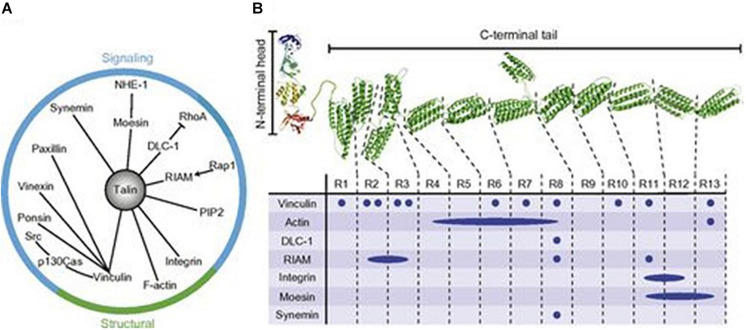
Talin structure and binding partners. **(A)** Relationship between talin and its signaling/structural partners. **(B)** Structure of the talin integrin-binding FERM domain head and mechanosensitive rod domain, with binding site locations. PIP2, phosphatidylinositol 4,5-bisphosphate. Adapted from Ref. (18). Copyright 1987 John Wiley & Sons – Journals. Published by Federation of American Societies for Experimental Biology. All rights reserved.

**FIGURE 11:**
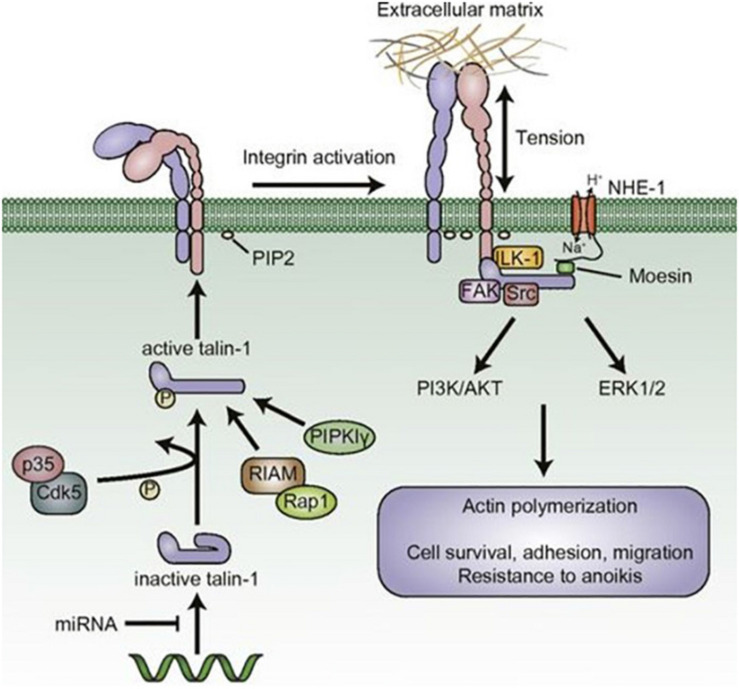
The role of talin in malignant phenotypes. Adapted from Ref. (18). Copyright 1987 John Wiley & Sons—Journals. Published by Federation of American Societies for Experimental Biology. All rights reserved.

In the study of Laberandie et al. (23), also cancer-associated fibroblasts (CAFs) can promote cancer invasion and metastases. According to this study, the fibroblast physical traction on cancer cells supports the invasive. Force transmission is mediated by a heterophilic adhesion involving N-cadherin at the CAF membrane and E-cadherin at the cancer cell membrane enabling cell adhesion, migration, and invasion (Figure 9). So many changes in cell–matrix adhesions are involved in the progression process from *in situ* to invasive carcinoma [for example, expression of E-cadherin calcium dependents is reduced in carcinomas (24)], and every step is strictly regulated by tumor DNA configuration that any epigenetic modification in the short run, induced by cosmetic medical instruments, seems quite unreasonable. Talin is another recently discovered large, plastic focal adhesion protein promoting interactions between actin cytoskeleton and integrins upon the cell membrane thus connecting the intracellular–extracellular environment (Figure 10). Haining et al. (25) demonstrated that Talin plays a key role in focal adhesion connecting intracellular networks with the extracellular matrix (ECM), via interaction between the actin cytoskeleton and membrane integrins. It focuses, in particular, on the mechanism of Talin compared to vinculin, providing a possible mechanism of cellular mechanotransduction, a process through which cells interpret and respond to physical stimuli. Talin has been shown to be significantly upregulated in the metastatic tissue of human prostate specimens compared with primary tumors, leading to greater adhesion, migration, and invasion (Figure 11). Now, we will concisely review the investigational state of the art of the clinical studies on this issue.

## Vibrational Therapy

Many studies show the benefits of full-body vibration therapy (WBV or whole-body vibration) in different diseases. Herrero et al. (26) assessed the effects of WBV on muscle activity, on the speed of blood flow in patients with spinal cord injury. WBV is effective in increasing leg, and restoring, muscle mass in patients with spinal injury. This observation might be a further work hypothesis in the treatment of muscle atrophy due to prolonged steroid administration or cancer cachexia. Cravenna claimed (27) the benefits of WBV therapy by a new instrument (Evocell) used in prostate cancer patients and females with postoperative or catheter-induced urinary incontinence and (28) without adverse oncological effects. Schönsteiner (29) reported the WBV-induced benefit in patients with polyneuropathy (common toxicity after chemotherapy) supplemented by massage and exercise. Pahl et al. (30) confirms the oncological safety of WBV administered to cancer-bearing patients.

## Shock Waves

Radial shock high-energy mechanical waves are generated by an electrode triggered in a chamber insulated by a rubber membrane in contact with the skin. They pass through the skin and spread radially into the body. Widely used to treat kidney stones, they are effective also in bone, joint, and tendon diseases, and even in cancer. The shock wave-spreading pathways are still unclear, but they produce mechanical cell rupture and increased cellular permeability with two basic mechanisms of action:

(1) Direct energy delivery enhanced by the reflectivity amplification either on soft (muscles and tendons) or hard (bones and calcifications) tissues and clusters.
(2) Indirect effect of “cavitation” on the cell membranes when the energy exceeds the elastic compliance of the tissue.

Both these action mechanisms activate the microvascular network and reduce the local inflammation, stimulating also osteogenesis in porotic or fractured bone. Two cell lines were experimentally treated with SW, one cancerized (CAKI-2) and the other normal (HK-2 and HRE): shock waves were highly detrimental for tumor cells rather than normal ones (31).

## Ultrasound

Ultrasound is a vibratory acoustic wave. Ultrasonic rays focus upon one target and spread harmlessly through the surrounding tissues. These are selectively absorbed accordingly with the emission frequency and tissue and organ density and also histomorphological structure. Temperature increases in the target areas, especially with high-intensity focused ultrasound (HIFU). HIFU have been experimentally used to treat pancreatic cancer (a highly lethal chemoresistant neoplasm), with encouraging results in terms of surrogate endpoint (life quality palliation HIFU) administration in combination with systemic chemotherapy, reducing the abdominal pain offering survival benefits and a few low-grade complications (32). In his systematic review, Cordeiro et al. (33) examined the effectiveness of HIFU as a primary procedure in 31 published papers plus two studies as a cancer rescue treatment. Most of the treated patients had localized prostate cancer, with an average age of 64 to 72 years. After 5 years, the survival rate ranged from 61.2 to 95% and after 7/8 years, it ranged from 69 to 84%. The complications of HIFU in the urogenital district were moderate. In neuroaesthetics, HIFU is promoted to reshape body fat tissue for aesthetic purposes, and so far, no adverse events have been reported in cancer patients, by our anecdotal experience.

Even if further specific systematic investigation is lacking, considering that HIFU is successfully endorsed to locally destroy cancer cells in the clinical setting, we suppose that indication of the same energy for cosmetic purposes is safe. As to low power ultrasounds, their safety is testified by the diagnostic use in obstetrics, gynecology, and with higher acoustic intensity, for remodeling subcutaneous fat (34).

## Laser and Pulsed Light

Laser and pulsed light (IPL) systems, widely used in aesthetics and dermatology, are considered fairly safe. However, the literature reports some possible side effects (35).

• Edema,
• Flittena or blisters from burn,
• Hypopigmentation,
• Hyperpigmentation,
• Scarring.

All laser and IPL systems use non-ionizing radiation that do not interfere with DNA. Studies denied any cause–effect relationship between laser aesthetic use and cancers (35). Aesthetically, there is a wide use of pulsed light and laser for permanent removal of the hair in hypertrichosis and hirsutism: their heating effect narrows the hair output ducts, preventing their shaft to exit and, in the long run, inducing bulb atrophy but not death.

## Radiofrequency

Radiofrequency is used in Wi-Fi systems, radio, television, and microwave ovens. The electromedical and cosmetical devices work with energies in the range of 400 kHz, with the following different delivery systems:

• **Monopolar radiofrequency:** it is made of two handles that generate opposite negative and positive energy, overheating the trespassed tissues down to the deep layers of the dermis and hypoderma. The body and the face are the usual targets of these instruments.
• **Bipolar resistive radiofrequency:** it delivers electric charges through two electrodes combined in the same handle. A higher localized thermal concentration is, thus, achieved.
• **Phototherapy or photoporation:** the absorbed electromagnetic energy induces different effects depending on the wavelength and can help low-molecular weight cosmeceuticals to pass across the skin barrier.
• **The capacitive radiofrequency:** works deep into the tissues through the placement of insulated electrodes, exciting the interstitial water molecules with heating effect.
• **The resisting radiofrequency:** heats the tissues by electrodes; when delivered through the fat, greater thermal effect induces lipolysis.

Generally speaking, the cosmetic final goal of the radiofrequencies is to reach the deep dermis and subacute, strengthening its texture, thickness, and elasticity. We did not find any, even if anecdotal, literature contribution about aesthetic radiofrequency administration to cancer patients. Our personal experience is that they effectively tighten the skin in the face, arms, and legs inducing a very good, even if temporary, rejuvenation effect; no untoward effects have been observed, with the warning of achieving an adequate hydration level of the skin before radiofrequency heat delivery. In the experimental setting, a 2-year study (36) exposed mice to 9 h/day mobile phone radiofrequencies (of 1,800 to 2.100 Mhz) and developed different cancer histotypes, but this protocol has nothing to do with the periodical cycles of cosmetic low-frequency radio apparatuses upon the human skin.

## Cryotherapy

Cryotherapy is widely used for cosmetic purposes in the following methods:

• **Localized cryotherapy** for single or multiple benign lesions. The sudden contact of the skin with low temperatures is painless and concentrated on single skin spots: it removes benign and malignant skin tumors and smoothens the skin surface improving translucency.
• **Cryosauna** is a vertical cabin in which liquid nitrogen is released in a gaseous state (-140°C for about 3 min). The patients stay for a few seconds into it, for a certain number of sessions, just the time to activate subcutaneous fat cell lipolysis and mesenchymal reticular and collagenous fiber turnover.
• **Cryotherapy associated with electroporation and chromotherapy** is addressed to localized areas of the body. The synchronous effects of electroporation currents or chromotherapy, with the sudden cooling of the target, enhance the mesenchymal cells turnover and reduce fat and cellulitis.

Cryotherapy is a historical weapon for the local treatment of cancers either of the skin or for the deep organs such as the liver, kidneys, etc. Freezing methods are safe: Zang (37) confirmed the role of hypothermia inhibiting the adhesion of cancer cells to endothelium and their migration. Kalamida D. et al. (38) screened the effect of fever-induced hyperthermia and mild hypothermia on human cancer cell in the lung cancer cell lines A549 and H1299, mcF7 breast adenocarcinoma, glioblastoma U87MG and T98G, prostate cancer DU145 and PC3, and cell lines of normal MRC5 fetal fibroblasts. After 3 days of exposure to different degrees of temperature, cellular vitality, apoptosis (caspase 9), and HSP90 expression were assessed with the following results:

• MRC5 fibroblasts are extremely sensitive to hyperthermia, but more resistant to hypothermia.
• T98G and A549 are thermo-tolerant and thermosensitive to varying degrees.
• In the U87MG thermal-sensitive cellular line, hyperthermia triggered caspase 9.
• In the thermo-tolerant T98G and A549 cell lines, caspase 9 levels decreased.
• Hyperthermia strongly induced HSP90 levels in T98G, while a sharp decrease was recorded in the PC3 and U87MG thermosensitive cell lines.
• Hyperthermia sensitized the cell lines of thermo-sensitive cancer to cisplatin and temozolomide, while its sensitizing effect decreased in thermo-tolerant cell lines.

On the basis of this investigation, we conclude that either moderate hyperthermia, or namely, hypothermia, reduces the vitality and/proliferation in all the above-described cell lines.

## Discussion and Conclusion

Our report, based on the literature screening and our personal long-standing clinical experience, was intended to verify whether the warning against the use of electromedical instruments in the cosmetic professional or medical cancer patient settings is consistent with evident oncological risks supported by experimental *in vitro/in vivo* dedicated studies or anecdotal clinical reports, or any other reasonable statement. The cancer cells are very vulnerable to almost all the physical existing energies due to their simplified antioxidant, defense, or DNA repair intrinsic potential. Most of the energies delivered by aesthetic electromedical instruments have been, or are currently, used with higher power to locally destroy cancer cells. The genetic and molecular mechanisms modulating cancer turnover investigated, so far, either in the induction or spreading or terminal phase, interact so finely and sequentially that any putative epigenetic interaction generated by this class of instruments seems gross and unrealistic. Our conclusions are in favor of moderate periodical use of cosmetic medical devices in patients bearing tumors, in any stage, like in healthy people.

Special consideration is dedicated to massage, manipulation, and pressure delivery upon the cytoskeleton of cancer cells that has proven to be sensitive to mechanical stress at least in some specific locally relapsing cancers such as osteosarcoma. Cancer mechanotransduction, a new investigational oncological profile, gives us some warnings about the due care when treating manually or with mechanical patients affected by malignant tumors, and let us hope in further new, instrumental, and molecular strategies to cope with cancer diffusion and metastasis. Obviously, during and after the aesthetic sessions, a medical judgment about the general individual trophic and local conditions of the skin and teguments, especially during chemo and oncological therapies, has to be carefully considered and adequately managed. Our strongly convinced opinion is that onco aesthetic care is, today, an extraordinary aid along the obscure tunnel of impending cancer diagnosis, treatments, and follow-up, giving a smart psychological and self-esteem support to the patients. It will be extremely helpful to avoid perceptions of discrimination and adequately cope with adverse destiny.

## Author Contributions

BP and MV: results and conclusions. LP and AM: materials and methods. VP: introduction. All authors contributed to the article and approved the submitted version.

## Conflict of Interest

The authors declare that the research was conducted in the absence of any commercial or financial relationships that could be construed as a potential conflict of interest.

## References

[1] Hanahan D Weinberg RA. Hallmarks of Cancer: the next generation. *Cell.* (2011) 144:646–74. 10.1016/j.cell.2011.02.013 21376230

[2] Shen R Ye Y Chen L Yan Q Barsky SH Gao JX. Precancerous stem cells can serve as tumor vasculogenic progenitors. *PLoS One.* (2008) 3:e1652. 10.1371/journal.pone.0001652 18286204 PMC2242848

[3] Chu WM. Tumor necrosis factor. *Cancer Lett.* (2013) 328:222–5. 23085193 10.1016/j.canlet.2012.10.014 PMC3732748

[4] Lou J Lucas R Grau GE. Pathogenesis of cerebral malaria: recent experimental data and possible applications for humans. *Clin Microbiol Rev.* (2001) 14:810–20. 10.1128/CMR.14.4.810-820.2001 11585786 PMC89004

[5] Institute of Medicine (US) Committee on Assessing Interactions Among Social, Behavioral, and Genetic Factors in Health, Hernandez LM Blazer DG. *Genes, Behavior, and the Social Environment: Moving Beyond the Nature/Nurture Debate.* Washington, DC: National Academies Press. (2006). 10.1128/cmr.14.4.810-820.2001 20669442

[6] Becker F van El CG Ibarreta D Zika E Hogarth S Borry P Genetic testing and common disorders in a public health framework: how to assess relevance and possibilities. Background document to the ESHG recommendations on genetic testing and common disorders. *Eur J Hum Genet.* (2011) 19 (Suppl. 1):S6–44. 21412252 10.1038/ejhg.2010.249 PMC3327518

[7] Moreno-Smith M Lutgendorf SK Sood AK. Impact of stress on cancer metastasis. *Future Oncol.* (2010) 6:1863–81. 21142861 10.2217/fon.10.142 PMC3037818

[8] Barre PV Padmaja G Rana S Tiamongla Stress and quality of life in cancer patients: medical and psychological intervention. *Indian J Psychol Med.* (2018) 40:232–8. 10.4103/ijpsym.ijpsym_512_17 29875530 PMC5968644

[9] Denaro N Tomasello L Russi EG. Cancer and stress: what’s matter? From epidemiology: the psychologist and oncologist point of view. *J Cancer Ther Res.* (2014) 3:6.

[10] Di Mattei VE Carnelli L Taranto P Bernardi M Brombin C Cugnata F “Health in the Mirror”: an unconventional approach to unmet psychological needs in oncology. *Front Psychol.* (2017) 8:1633. 10.3389/fpsyg.2017.01633 28983271 PMC5613306

[11] Dorr Goold S Lipkin M Jr. The doctor-patient relationship: challenges, opportunities, and strategies. *J Gen Intern Med.* (1999) 14 (Suppl. 1):S26–33. 9933492 10.1046/j.1525-1497.1999.00267.x PMC1496871

[12] Molassiotis A Fernández-Ortega P Pud D Ozden G Scott JA Panteli V Use of complementary and alternative medicine in cancer patients: a European survey. *Ann Oncol.* (2005) 16:655–63. 10.1093/annonc/mdi110 15699021

[13] Verhoef MJ Balneaves LG Boon HS Vroegindewey A. Reasons for and characteristics associated with complementary and alternative medicine use among adult cancer patients: a systematic review. *Integr Cancer Ther.* (2005) 4:274–86. 10.1177/1534735405282361 16282504

[14] Kristoffersen AE Norheim AJ Fønnebø VM. Complementary and alternative medicine use among norwegian cancer survivors: gender-specific prevalence and associations for use. *Evid Based Comp Altern Med.* (2013) 2013:318781. 10.1155/2013/318781 23606877 PMC3625602

[15] Boehm K Büssing A Ostermann T. Aromatherapy as an adjuvant treatment in cancer care – a descriptive systematic review. *Afr J Trad Comp Alternat Med.* (2012) 9:503–18. 10.4314/ajtcam.v9i4.7 23983386 PMC3746639

[16] Stylianopoulos T. The solid mechanics of cancer and strategies for improved therapy. *J Biomech Eng.* (2017) 139:021004 10.1115/1.4034991 PMC5248974 27760260

[17] Wang JY Wu PK Chen PCH Yen CC Hung GY Chen CF Manipulation therapy prior to diagnosis induced primary osteosarcoma metastasis – from clinical to basic research. *PLoS One.* (2014) 9:e96571. 10.1371/journal.pone.0096571 24804772 PMC4013034

[18] Babahosseini H Roberts PC Schmelz EM Agah M. Roles of bioactive sphingolipid metabolites in ovarian cancer cell biomechanics. *Annu Int Conf IEEE Eng Med Biol Soc.* (2012) 2012:2436–9. 23366417 10.1109/EMBC.2012.6346456

[19] Brossel R Yahi A David S Velasquez LM Guinebretière JM. Mechanical signals inhibit growth of a grafted tumor *in vivo*: proof of concept. *PLoS One.* (2016) 11:e0152885. 10.1371/journal.pone.0152885 27100674 PMC4839666

[20] Hui KK Marina O Liu J Rosen BR Kwong KK. Acupuncture, the limbic system, and the anticorrelated networks of the brain. *Auton Neurosci.* (2010) 157:81–90. 10.1016/j.autneu.2010.03.022 20494627 PMC3754836

[21] Tsai T-J Chung U-L Chang C-J Wang H-H. Influence of religious beliefs on the health of cancer patients. *Asian Pac J Cancer Prev.* (2016) 17:2315–20. 10.7314/apjcp.2016.17.4.2315 27221937

[22] Craig DH Owen CR Conway WC Walsh MF Downey C Basson MD. Colchicine inhibits pressure-induced tumor cell implantation within surgical wounds and enhances tumor-free survival in mice. *J Clin Invest.* (2008) 118:3170–80. 10.1172/jci34279 18704196 PMC2515382

[23] Labernadie A Kato T Brugués A Serra-Picamal X Derzsi S Arwert E. A mechanically active heterotypic E-cadherin/N-cadherin adhesion enables fibroblasts to drive cancer cell invasion. *Nat Cell Biol.* (2017) 19:224–37. 10.1038/ncb3478 28218910 PMC5831988

[24] Byers SWSW Sommers CLCL Hoxter BB Mercurio AM Tozeren AA. Role of E-cadherin in the response of tumor cell aggregates to lymphatic, venous and arterial flow: measurement of cell-cell adhesion strength. *J Cell Sci.* (1995) 108 (Pt 5):2053–64. 7657723 10.1242/jcs.108.5.2053

[25] Haining AW Lieberthal TJ Del Río Hernández A. Talin: a mechanosensitive molecule in health and disease. *FASEB J.* (2016) 30:2073–85. 10.1096/fj.201500080r 27252130

[26] Herrero AJ Menéndez H Gil L Martín J Martín T García-López D Effects of whole-body vibration on blood flow and neuromuscular activity in spinal cord injury. *Spinal Cord.* (2011) 49:554–9. 10.1038/sc.2010.151 21042329

[27] Crevenna R Cenik F Margreiter M Marhold M Sedghi Komanadj T Keilani M. Whole body vibration therapy on a treatment bed as additional means to treat postprostatectomy urinary incontinenceGanzkörpervibration auf einer Therapieliege als zusätzliche Therapiemöglichkeit bei Inkontinenz nach Prostataoperation. *Wien Med Wochenschr.* (2016) 167:139–41. 10.1007/s10354-016-0469-7 27342596

[28] Farzinmehr A Moezy A Koohpayehzadeh J Kashanian M. A comparative study of whole body vibration training and pelvic floor muscle training on women’s stress urinary incontinence: three- month follow- up. *J Fam Reprod Heal.* (2015) 9:147–54. PMC4818376 27047560

[29] Schönsteiner SS Bauder Mißbach H Benner A Mack S Hamel T Orth M A randomized exploratory phase 2 study in patients with chemotherapy-related peripheral neuropathy evaluating whole-body vibration training as adjunct to an integrated program including massage, passive mobilization and physical exercises. *Exp Hematol Oncol.* (2017) 6:5. 10.1186/s40164-017-0065-6 28194306 PMC5297221

[30] Pahl A Wehrle A Kneis S Gollhofer A Bertz H. Feasibility of whole body vibration during intensive chemotherapy in patients with hematological malignancies – a randomized controlled pilot study. *BMC Cancer.* (2018) 18:920. 10.1186/s12885-018-4813-8 30253746 PMC6156963

[31] Li D Pellegrino A Hallack A Petrinic N Jérusalem A Cleveland RO. Response of single cells to shock waves and numerically optimized waveforms for cancer therapy. *Biophys J.* (2018) 114:1433–9. 10.1016/j.bpj.2017.09.042 29590600 PMC5883951

[32] Wu F. High intensity focused ultrasound: a noninvasive therapy or locally advanced pancreatic cancer. *World J Gastroenterol.* (2014) 20:16480–8. 10.3748/wjg.v20.i44.16480 25469016 PMC4248191

[33] Cordeiro ER Cathelineau X Thüroff S Marberger M Crouzet S de la Rosette JJ. High-intensity focused ultrasound (HIFU) for definitive treatment of prostate cancer. *BJU Int.* (2012) 110:1228–42. 10.1111/j.1464-410x.2012.11262.x 22672199

[34] Izadifar Z Babyn P Chapman D. Mechanical and biological effects of ultrasound: a review of present knowledge. *Ultrasound Med Biol.* (2017) 43:1085–104. 10.1016/j.ultrasmedbio.2017.01.023 28342566

[35] Ash C Town G Whittall R Tooze L Phillips J. Lasers and intense pulsed light (IPL) association with cancerous lesions. *Lasers Med Sci.* (2017) 32:1927–33. 10.1007/s10103-017-2310-y 28884244 PMC5653718

[36] Wyde ME Horn TL Capstick MH Ladbury JM Koepke G Wilson PF Effect of cell phone radiofrequency radiation on body temperature in rodents: pilot studies of the National Toxicology Program’s reverberation chamber exposure system. *Bioelectromagnetics.* (2018) 39:190–9. 10.1002/bem.22116 29537695

[37] Zhang X Lv YG Chen GB Zou Y Lin CW Yang L Effect of mild hypothermia on breast cancer cells adhesion and migration. *Biosci Trends.* (2012) 6:313–24. 10.5582/bst.2012.v6.6.313 23337791

[38] Kalamida D Karagounis IV Mitrakas A Kalamida S Giatromanolaki A Koukourakis MI. Fever-range hyperthermia vs. hypothermia effect on cancer cell viability, proliferation and HSP90 expression. *PLoS One.* (2015) 10:e0116021. 10.1371/journal.pone.0116021 25635828 PMC4312095

